# Retraction: Splenic Injury After Colonoscopy in a 55-Year-Old Female Patient

**DOI:** 10.7759/cureus.r80

**Published:** 2023-12-13

**Authors:** Ilias Galanis, Magdalini Simou

**Affiliations:** 1 Second Department of Surgery, Evangelismos General Hospital, Athens, GRC

This article has been retracted at the request of the authors due to myriad errors discovered after publication:

 

1. The patient underwent a colonoscopy approximately five weeks (and not five days, as stated in the article) before the diagnosis of splenic injury.
2. After the colonoscopy, but before the diagnosis of splenic trauma, the patient had an accident (fall from a ladder) which constitutes a far more probable cause of the splenic injury than the colonoscopy procedure.
3. The patient was on anticoagulant therapy, which is not mentioned in the article.

As a result of the above, the authors now believe that this is a regular case of traumatic splenic injury rather than a rare case of splenic injury due to colonoscopy. The authors take full responsibility for, and deeply regret, the above errors and omissions.

